# Enhanced healing of wounds that responded poorly to silver dressing by copper wound dressings: Prospective single arm treatment study

**DOI:** 10.1002/hsr2.1816

**Published:** 2024-01-14

**Authors:** Oxana Gorel, Mozna Hamuda, Ilana Feldman, Irit Kucyn‐Gabovich

**Affiliations:** ^1^ Loewenstein Rehabilitation Center Ra'anana Israel

**Keywords:** amputations, noninfected wounds, silver dressing, copper dressing, surgery wounds, wound management

## Abstract

**Background and Aims:**

Dressings containing silver ions are an accepted and common option for wound treatment. However, some wounds fail to heal at the desired rate despite optimal management. The aim of the study was to examine the effect of copper dressings in noninfected wounds.

**Methods:**

The study included 20 patients aged 18–85 years with 2–30 cm^2^ noninfected wounds treated for 17–41 days with silver wound dressings that failed to reduce by >50% the wound size, who were then treated with copper dressings. Ten patients were diabetics, 10 suffered from hypertension, and six suffered from peripheral vascular disease (PVD). Two patients suffered from two wounds. Most were amputation wounds below the knee.

**Results:**

Five patients dropped out from the study due to complications not related to the wound. The mean period of silver and copper dressings treatment was 25.6 and 29.6 days, respectively (*p* = 0.25; *t* test). None of the wounds became infected. Comparing a period of 25 days, during the copper dressings treatment, the mean wound area reduction was ~2.4 times higher than during the silver dressing treatment, 87.35 ± 22.4% versus 37.02 ± 25.11% (mean ± SD; *p* < 0.001; paired *t* test), respectively. The average decline during the silver and copper treatments were 1.2% and 2.14% per day (*p* = 0.002; multiple regression analysis), respectively.

**Conclusions:**

The enhanced wound healing process observed with the copper dressings may be explained by the integral role of copper throughout all physiological skin repair processes. Silver in contrast has no physiological role in wound healing. The results of our study confirm case reports showing enhanced wound healing of hard‐to‐heal wounds with copper dressings, both of infected and noninfected wounds. Taken together, the results of the current study support the hypothesis that the application of copper dressings in situ for noninfected wounds results in the stimulation of the wound healing processes, as opposed to silver dressings.

## 1 INTRODUCTION

The global population's growth and aging contribute to a heightened incidence of wounds, including those that become infected.^1^ Infections within wounds pose a substantial hindrance to the healing process, significantly impeding the transition from the inflammatory phase to the proliferative phase. This obstacle often leads to the persistence of chronic wounds.^2^

Management of wound infections has been increasingly complex as the prevalence of antibiotic‐resistant microorganisms continues to rise.^3^ The formation of microbial biofilms within wounds not only decreases the effectiveness of antimicrobial treatments but can even thwart their efficacy entirely. The consequences of wound infections place a considerable burden on both patients and healthcare systems alike.^4^, ^5^ Safeguarding wounds against pathogens stands as a pivotal clinical practice in effective wound management. In this regard, wound dressings assume a critical role in not only preventing but also treating wound infections.^6^

The utilization of antimicrobial wound dressings has witnessed a steady rise in their application for both the treatment of infected wounds and the prevention of wound infection.^7^ Most commercially available antimicrobial wound dressings contain a variation of silver as the active ingredient. Silver has wide‐spectrum antimicrobial efficacy but there is a growing number of reports indicating that silver may be detrimental to the wound healing physiological processes.^8^, ^9^, ^10^, ^11^, ^12^

There are two key properties of copper that make it a very attractive option for the management of wounds. First, copper is a potent wide‐spectrum biocide.^13^, ^14^ The detrimental effects inflicted upon these microorganisms stem from a range of indiscriminate mechanisms. These encompass the permeabilization of their plasma membranes, the peroxidation of membrane lipids, impairment to their nucleic acids, and the disruption of the assembly and functionality of intracellular proteins.^15^ The intricate web of multisite, nonspecific copper‐induced damages poses a considerable challenge for microorganisms to evolve a resistance to copper, resulting in a notably low occurrence of copper‐tolerant microorganisms.^15^, ^16^ Second, and most importantly, copper is an essential trace mineral required for efficient wound healing.^17^, ^18^ For example, processes such as angiogenesis, proliferation of dermal fibroblasts, the enhanced expression of collagen and elastin fiber secretion by these fibroblasts, and the interlinking of extracellular matrix (ECM) proteins, all require copper.

Recently novel wound dressings impregnated with copper microparticles have been approved for the clinical management of acute and chronic wounds by the United States, European, and Israeli regulatory bodies. These dressings possess potent antimicrobial efficacy,^19^ and several reports indicate their capacity to enhance wound healing, especially of diabetic ulcers.^20^, ^21^, ^22^

In our rehabilitation center, part of the standard of care of acute and chronic wounds is the use of silver dressings, mainly to protect the wounds from infection. However, in view of the growing evidence that silver dressings may impede the wound healing processes, it was decided to examine the effect of the copper dressings in noninfected wounds treated with silver dressings, but in whom the wounds were not healing or the progression of the wound healing were slow. The current study was devised to examine the efficiency of copper dressing to improve wound healing among patients admitted to a rehabilitation hospital. The goal of this study was to evaluate the effect of the copper dressings in noninfected wounds treated with silver dressings, but in whom the wounds were not healing or the progression of the wound healing were slow. To the best of our knowledge, similar investigations have not been reported in the literature for patients in a rehabilitation setting.

## 2 MATERIAL AND METHODS

### 2.1 Study group

The study group included twenty 18–85 years old patients with pressure ulcers, diabetic ulcers, trauma wounds, or postoperation wounds with wound areas of 2–30 cm^2^. Out of the 20 patients that were recruited, 15 patients completed the study protocol and their data were analyzed. Three patients received antibiotics and two were transferred to a different hospital shortly after their recruitment, all five due to complications not related to the study or the wound. Table 1 summarizes the general characteristics of the 15 patients who finished the study. Ten of the patients were diabetics (mean ± SD % HbA1c of 6.8 ± 0.99 (CI = 0.02)); two of which their diabetes was not fully successfully controlled. Ten of the patients suffered from hypertension and six from peripheral arterial disease (PVD). Nine were smokers and two were receiving immunosuppression medication.

**Table 1:** General baseline characteristics of the patients.

Parameter	Unit	Value
Age, years	Mean ± SD	56.6 ± 11.8
Weight, Kg	Mean ± SD	82.3 ± 17.1
Sex, male	*n* (%)	12 (80)
Type 2 diabetes	*n* (%)	10 (67)
Control of diabetes^a^	*n* (%)	8 (53)
Renal disease	*n* (%)	1 (7)
Hypertension	*n* (%)	10 (67)
Cardiovascular disease	*n* (%)	3 (20)
Peripheral arterial disease	*n* (%)	6 (40)
Venous disease	*n* (%)	2 (13)
Immunosuppression medication	*n* (%)	2 (13)
Smokers	*n* (%)	9 (60)
HbA1c, % (diabetic patients)	Mean ± SD	6.8 ± 0.99
Hemoglobin, g/dL	Mean ± SD	13.2 ± 1.52
White blood cells count, n/µL	Mean ± SD	7.68 ± 1.25
Creatine, µmol/L	Mean ± SD	0.92 ± 0.23

^a^ During the whole trial there were no fluctuations above 200 mg/dL glucose.

All of these patients were treated with Silvercel Hydro (Systagenix) or Aquacel Ag (Convatec), hereafter referred to as “silver dressings,” for at least 3 weeks, but in all of them their wounds did not show clinical improvement or a reduction of at least 50% of the wound area during the silver dressing treatment. All of these patients met the other inclusion and exclusion criteria (Table 2). They signed the informed consent form and were recruited to the study. Their wounds were then treated with Antimicrobial Wound Dressings with Copper (MedCu Technologies Ltd.), hereafter referred to as “copper dressings,” for at least 3 weeks. In case there was a clear improvement during the copper dressing treatment, the wounds continued to be treated with the copper dressings until wound closure.

**Table 2:** Inclusion/exclusion criteria.

Inclusion criteria	Exclusion criteria
Males and females aged between 18 and 85 years	A clinically significant active or unstable cardiac, gastrointestinal, endocrine, neurological, liver, or kidney disease
All type of wounds	Psychiatric condition
In case of post‐op wounds, at least three weeks post‐op	Active participation in an investigational trial within 30 days of the screening visit
Wound area between 2 to 30 cm^2^	History of allergic reactions attributed to copper
Noninfected wounds	Patient with known allergy to at least three drugs or other substances
Wounds that do not show significant healing when treated with silver dressings (reduction of wound size of less than 50% in 3 weeks)	Any chronic or acute condition susceptible of interfering with the evaluation of the wound dressing effect
Not undergoing any systemic or topical antibiotic treatment for the wound for a week before enrollment in the study	Individuals using and need to continue use any type of topical agents in or on the wound
Be available for the entire study period, and ability and willingness to adhere to the requirements of the study	Females who are pregnant, lactating, of child‐bearing potential
Having at least moderate blood perfusion into the affected limb as defined by palpable pulses (Dorsalis Pedis and/or Tibialis Posterior, unequivocally palpable). If no pulse is clearly present in vascular lab tests Ankle Brachial Index (ABI) should be 0.6< or if ABI > 1.3, then toe pressure of > 50 mmHg	Any form of substance abuse (including drug or alcohol abuse, excluding cannabis), psychiatric disorder, or any chronic condition susceptible, in the opinion of the investigator, of interfering with the conduct of the study
The patient is able and eligible to sign written informed consent and participate in the study	Fertile female subjects who are not willing to use an acceptable method of contraception during the study
	Subjects who are likely to be noncompliant or uncooperative during the study
	Wound‐related parameters: The size of the wounds is reduced by more than 50% during 3 weeks of using silver dressing. Wounds in which local pressure cannot be avoided due to protruding bone, or any other complex rehabilitation procedure. Infected or necrotic wounds. Wounds that are considered to necessitate debridement in the operation room during the study. Wounds that necessitate antibiotic treatment or needed antibiotic treatment 1 week before the trial. Wound with tunnels of more than 3 cm.
	Lab parameters: Hemoglobin below 7.0 g/dL White blood cells count > 14,000/μL Albumin <2.5 g/dL

### 2.2 Assessments and procedures

The silver or copper dressings were pretrimmed to an adequate size and shape based on the wound size and form. After they were applied onto the wound, a secondary dressing was applied on top of the dressings to hold them in place. The silver or copper dressings were replaced every 2–4 days, depending on the amount of wound exudate. The area of the wounds was measured routinely every 7 days during the study by using the wound imaging artificial intelligence system (Tissue Analytics; https://www.tissue-analytics.com/). All wounds and surrounding tissues were carefully cleaned and irrigated with sterile sodium chloride solution before taking an image of the wound. The percent reductions as compared with the area of the wounds at the commencement of the treatment with silver or copper dressings were determined using the following formula:

$$\text{Proportion}\;\text{of}\;\text{wound}\;\text{closure}=\frac{\text{Initial}\;\text{wound}\;\text{area}\;\left({\mathrm{cm}}^{2}\right)–\text{wound}\;\text{area}\;\text{after}\;\text{treatment}\;\left({\mathrm{cm}}^{2}\right)}{\;\text{Initial}\;\text{wound}\;\text{area}\;\left({\mathrm{cm}}^{2}\right)}.$$

If the wound became infected and the patient received antibiotics, the patient was dropped from the trial. The number of infections and adverse reactions were recorded.

The study was approved by the Lowenstein Rehabilitation Center IRB (approval # 0002‐20‐LOE) and was registered in ClinicalTrials.gov Protocol Registration and Results System (# NCT04634838; 002‐20‐LOE).

### 2.3 Statistics

Paired *t* tests were performed to analyze the impact of two Arms (different treatment periods) on the wound size/area. Our primary objective was to evaluate the disparity between the wound size when the particular treatment was initiated and the endpoint of the wound during that treatment by comparing the change in size, referred to as delta, between “day 0” and “day 25.” We also employed multiple regression analysis to examine the influence of two treatment group (silver vs. copper) on the wound size/area over time. Our objective was to assess the disparity in the rate of wound closure by comparing the slopes of the regression lines between the two treatments. A significance level of 0.05 was selected to determine the statistical significance of the results and all tests were two‐sided. Analyses were performed using JMP® Pro, Version 16.

## 3 RESULTS

Two patients suffered from two wounds each, and thus 17 wounds were analyzed. All wounds were noninfected as determined by clinical examination. Table 3 describes the wound parameters at the start of the study. Nine of the wounds were post‐op wounds following amputation below the knee, two of which were following trauma. None of the wounds had protruding bones or necrotic tissue. The mean ± SD of the initial wound area was 8.57 ± 6.4, (CI = 0.1) based on the AI software determinations.

**Table 3:** Wound characteristics at the commencement of the study.

Parameter	
Type of wound, *n* (%):	
• Post‐op	10 (52.6)
• Trauma	4 (21.0)
• Diabetic foot ulcer	2 (10.5)
• Pressure ulcer	1 (5.4)
• Other	2 (10.5)
Location, *n* (%):	
• Dorsal foot	1 (5.9)
• Pedal foot	2 (11.8)
• Leg below knee	13 (76.4)
• Other	1 (5.9)
Necrotic tissue, *n* (%)	0 (0)
Protruding bones, *n* (%)	0 (0)
Initial wound area, cm^2^, mean (SD)	8.57 (6.4)
Initial wound perimeter, cm, mean (SD)	14.58 (6.73)

**Figure 1:**
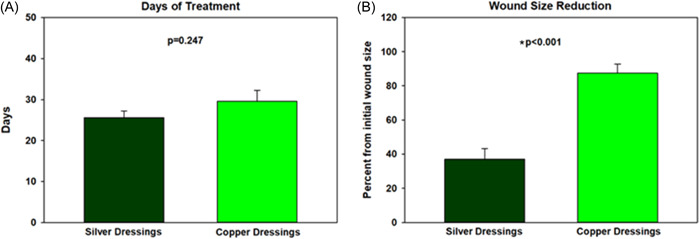
Average days of treatment with the silver and copper dressings (a) and average precent of wound size reduction during the respective treatments (b). The bars show the mean and standard deviation.

**Figure 2:**
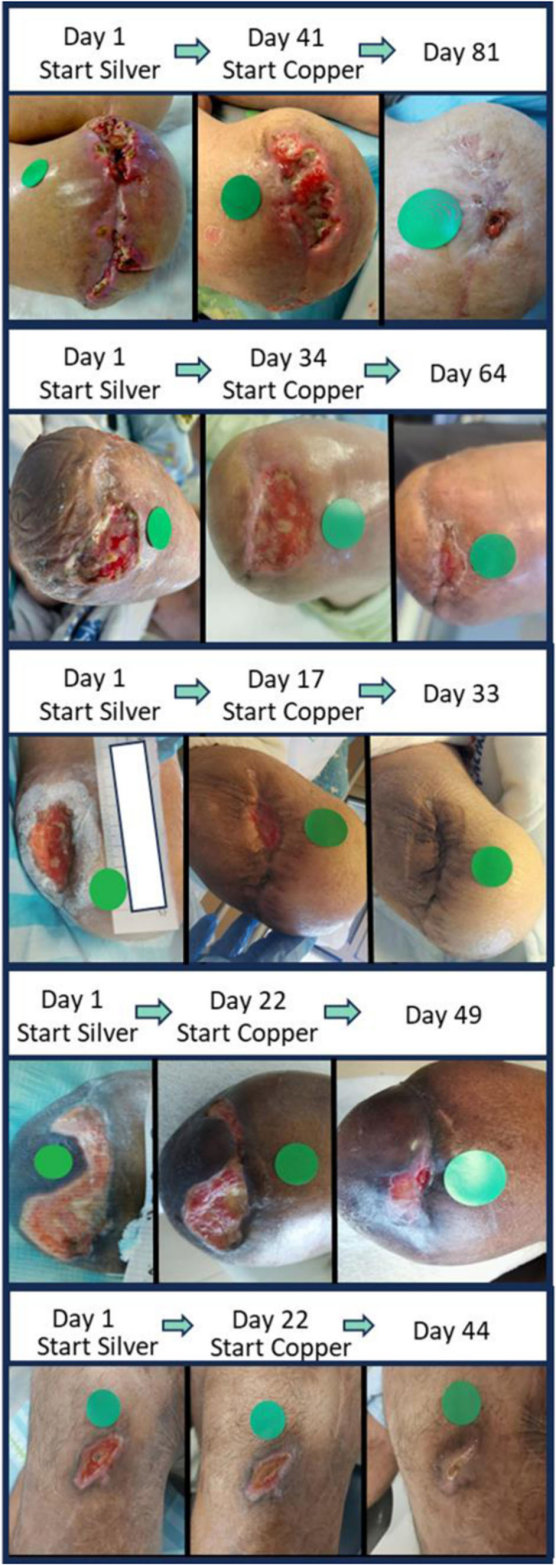
Representative pictures of wounds treated initially with silver dressings and then treated with copper dressings.

**Figure 3:**
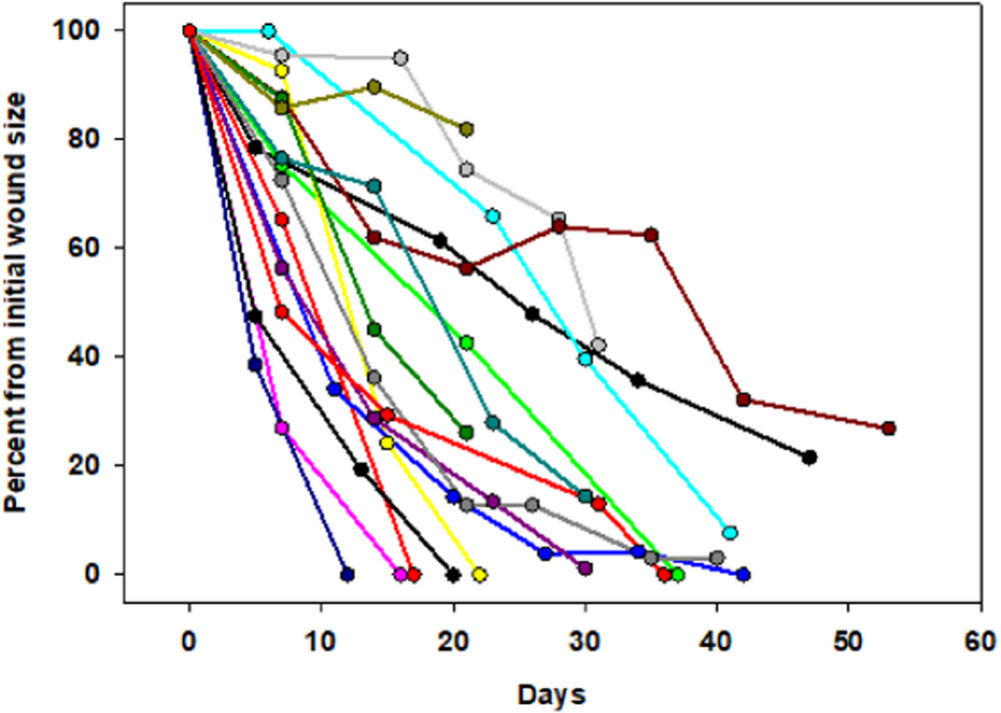
Wound healing progression. Each line represents the size of the wound per each patient after normalization to the start of the copper dressing use.

The period of silver and copper dressings treatment were similar, mean of 25.6 ± 6.6 (confidence interval [CI] = 0.1) and 29.6 ± 11.1 days (CI = 0.16) (mean ± SD; *p* = 0.21; *t* test), respectively (Table 4 and Figure 1A). Comparing a period of 25 days, the mean proportion wound area reduction was ~ 2.4 times higher during the copper dressing treatment than during the silver dressing treatment, 87.35 ± 22.4% (CI = 0.37) versus 37.02 ± 25.11% (CI = 0.33) (mean ± SD; *p* < 0.001; paired *t* test; Figure 1B), respectively. Some representative examples are shown in Figure 2. It was found that the rate of decrease in wound size was significantly different between the two treatment groups. While the average decline during the silver treatment was 1.2% per day, during the copper treatment the average decline per day was 2.14% (multiple regression; *p* = 0.002). Ten out of the 15 patients closed their wounds following the copper dressings treatment. Some of the 15 patients responded immediately to the exposure to the copper dressings; six of them were diabetics. Some took them longer to close the wounds (Figure 3). Out of the diabetic patients, six closed the wounds completely.

**Table 4:** Infection episodes during the silver and copper periods of treatment.

Treatment	Period of treatment (Days)		Infection episodes
	Mean ± SD	Range	#
Silver	25.59 ± 6.6	17–41	3
Copper	29.59 ± 11.15	12–46	0
*p* Value	0.21^a^	‐	0.07^b^

^a^ *t* test for unequal variances.

^b^ *χ* ^2^.

## 4 DISCUSSION

While silver and copper have wide spectrum antimicrobial properties, as opposed to silver, copper is a trace mineral essential for the normal function of all human tissues.^23^ In wounds, copper is involved in many of the wound healing processes, such as angiogenesis, stimulation of secretion of fibrinogen, elastin and collagen by dermal fibroblasts, induction of cell proliferation and re‐epithelization, and migration of skin and stem cells.^24^, ^25^, ^26^, ^27^, ^28^, ^29^ It has been stipulated that in diabetic and other hard‐to‐heal stagnated wounds part of their incapacity to heal or the reason they heal slowly is the low systemic copper that reaches the wound through the integumentary system,^17^ and that the external application of dressings that elute copper ions onto the wound may stimulate the stagnated healing processes.^17^, ^20^ The capacity of copper eluted from copper‐containing dressings to stimulate noninfected wounds as opposed to controlled dressings without an active ingredient or silver‐containing dressings was clearly demonstrated in wounds elicited in genetically engineered mice.^25^ This was further substantiated by Das et al.,^30^ who demonstrated in wild mice that exogenous and endogenous Cu promote wound healing through the endothelial antioxidant‐1 (Atox 1) cytosolic Cu chaperone. Meaningfully, the capacity of copper dressings to stimulate wound healing of noninfected stagnated wounds in diabetic patients was also demonstrated.^22^ Several clinical case reports that demonstrated enhanced wound healing of hard‐to‐heal wounds by copper dressings in patients suffering from other etiologies were also reported.^20^, ^21^, ^31^ Coger et al.^32^ showed that following wounding there is almost a 40% increase in the copper concentration in the wound in normal healthy rats, indicating that the normal physiological response to wounding, among many processes, is the increased delivery of copper to the wounds. In the wound, numerous intricately balanced mechanisms that drive wound healing and repair rely significantly on their interactions with copper.^17^, ^18^, ^33^, ^34^ This encompasses various essential components: platelet‐derived growth factor (PDGF), playing a crucial role in the hemostasis phase of wound healing; vascular endothelial growth factor (VEGF) and angiogenin, pivotal growth factors stimulating angiogenesis, a critical process during the proliferation phase; dermal fibroblasts actively secreting collagens (types I, II, and V), Heat Shock Protein 47 (HSP‐47), and elastin fiber constituents (elastin, fibrillins) throughout the proliferation and remodeling phases; the activity of Lysyl oxidase (LOX) essential for efficient ECM protein cross‐linking between elastin and collagen; maintenance of stabilized skin ECM postformation; differentiation‐induced modulation of integrins by keratinocytes during the remodeling phase; and participation of major protease groups including matrix metalloproteinases (MMPs, primarily MMP‐1, MMP‐2, MMP‐8, MMP‐9) and serine proteases (human neutrophil elastase, HNE) crucial for the wound healing process. It is therefore unsurprising that wound closure is delayed due to copper chelation.^30^ Interestingly, Yadav et al.^35^ found that in diabetic patients with foot ulcers the serum concentration of copper was lower than in diabetic patients without ulcers. This is in accordance with the notion that a small scratch or wound in a diabetic patient with low copper levels may not heal and become a nonhealing wound, while in patients and individuals with high serum copper concentration the scratch/wound is healed.^17^

In the current study, we applied copper dressings in a variety of wounds, mostly in post‐op amputation wounds below the knee. Most of the patients were diabetics, most suffered from hypertension, and many from peripheral arterial disease. Nine out of the 15 patients were smokers, three suffered from cardiovascular disease, two from venous disease or renal disease, and two were taking immunosuppression medication. In all of these patients, the wound‐healing process seemed to be slow when they were managed with silver dressings. This is in accordance with several studies showing that silver may impede the wound healing processes and their benefit in managing wounds and especially of noninfected wounds is questionable.^8^, ^9^, ^10^, ^11^ In contrast, the application of the copper dressings had a very positive effect on the wound healing, leading to the eventual closure of the wounds in most of the patients without any adverse effects. No clear correlation between the rate of wound closure and the effect of the dressings could be established, mainly due to the low number of patients studied. Larger studies should be conducted to better decipher why some wounds and/or patients respond better than others to the effect of the copper dressings. While we planned to have a larger number of patients included in the study, we decided to stop the study and manage the wound of our patients only with copper dressings, as we felt it was not ethical to treat our patients with the silver dressings while we were already convinced that the copper dressings had an excellent effect and helped heal the wounds significantly better than the silver dressings.

## 5 CONCLUSION

The results of this study confirm other clinical case reports showing enhanced wound healing of hard‐to‐heal wounds with copper dressings, both of infected and noninfected wounds. Further studies should be conducted to further support the clinical benefit of using copper dressings instead of silver dressings for the management of acute and chronic wounds. Taken together, the results of the current study clearly support the hypothesis that application of copper dressings in situ onto noninfected wounds results in the stimulation of the wound healing processes in a panel of etiologies.

## AUTHOR CONTRIBUTIONS

**Oxana Gorel**: Conceptualization; data curation; formal analysis; investigation; project administration; supervision; writing—review & editing. **Irit Kucyn‐Gabovich**: Conceptualization; methodology; project administration; data curation; writing. Monza Hamuda and Ilana Feldman were involved in the treatment and management of the patients' wounds. All authors have read and approved the final version of the manuscript.

## CONFLICT OF INTEREST STATEMENT

The authors declare no conflict of interest.

## TRANSPARENCY STATEMENT

The lead author Oxana Gorel affirms that this manuscript is an honest, accurate, and transparent account of the study being reported; that no important aspects of the study have been omitted; and that any discrepancies from the study as planned (and, if relevant, registered) have been explained.

## Data Availability

The data that support the findings of this study are available from the corresponding author upon reasonable request. O.G. had full access to all of the data in this study and takes complete responsibility for the integrity of the data and the accuracy of the data analysis. The individual participant data that underlie the results reported in this article, after deidentification, will be available upon email request from the corresponding author immediately after publication and ending 3 years following publication.
